# A biophysical model of kiwifruit (*Actinidia deliciosa*) berry development

**DOI:** 10.1093/jxb/ert317

**Published:** 2013-10-11

**Authors:** Alistair J. Hall, Peter E. H. Minchin, Michael J. Clearwater, Michel Génard

**Affiliations:** ^1^The New Zealand Institute for Plant & Food Research Limited (PFR), Private Bag 11600, Palmerston North 4442, New Zealand; ^2^PFR, 412 No 1 Road, RD 2, Te Puke 3182, New Zealand; ^3^Department of Biological Sciences, University of Waikato, Private Bag 3105, Hamilton 3240, New Zealand; ^4^INRA, UR 1115 Plantes et Systèmes de culture Horticoles, Domaine St Paul-Agroparc, F-84914 Avignon, France

**Keywords:** Fruit growth model, mass flow, osmotic pressure, pedicel, starch, transport, water.

## Abstract

A model of kiwifruit berry development is presented, building on the model of Fishman and Génard used for peach fruit. That model has been extended to incorporate a number of important features of kiwifruit growth. First, the kiwifruit berry is attached to the stem through a pedicel/receptacle complex which contributes significantly to the hydraulic resistance between the stem and the fruit, and this resistance changes considerably during the season. Second, much of the carbohydrate in kiwifruit berries is stored as starch until the fruit matures late in the season, when the starch hydrolyses to soluble sugars. This starch storage has a major effect on the osmotic potential of the fruit, so an existing model of kiwifruit starch dynamics was included in the model. Using previously published approaches, we also included elasticity and extended the modelling period to cover both the cell division and cell expansion phases of growth. The resulting model showed close simulation of field observations of fresh weight, dry matter, starch, and soluble solids in kiwifruit. Comparison with continuous measurements of fruit diameter confirmed that elasticity was needed to adequately simulate observed diurnal variation in fruit size. Sensitivity analyses suggested that the model is particularly sensitive to variation in inputs relating to water (stem water potential and the humidity of the air), and to parameters controlling cell expansion (cell wall extensibility). Some limitations in the model structure were identified, suggesting that a revised model including current apoplastic/symplastic concepts needs to be developed.

## Introduction

Fruit growth involves both phloem and xylem flow, transpiration from the fruit surface, and the internal processes of cell division, differentiation, metabolism, and catabolism. Final fruit size and composition are a result of the coupled import of water and carbon during growth. Carbohydrate, usually sucrose, arriving in the phloem is the major substrate for growth and storage, but is also osmotically active, thereby influencing phloem flow into the fruit. Inter-conversions in the chemical form and location of solutes also impact on the fruit water balance and growth via osmotic interactions. The external environment affects fruit transpiration as well as the phloem and xylem flows, so affects fruit size as well as fruit composition. Genotype differences also alter final fruit composition and fruit size. A process-based model of fruit growth is needed to provide integration of the known chemical and physical processes involved.

The aim of this study was to develop a physiological model of fruit development of kiwifruit, capable of simulating development from soon after bloom to maturity. The approach has required an extension of the peach growth model of Fishman and Génard (1998), to include a pedicel, multiple growth phases, and elasticity, which were not included in the original peach model that only applied to the post-stone-hardening phase of peach growth.

We have used the approach of Léchaudel *et al.* (2007) applied to mango fruit to include elasticity, and that of Liu *et al.* (2007) applied to tomato fruit to include both the cell division and cell expansion phases. Extension to multiple phases of growth was achieved by allowing the cell wall extensibility to vary through several orders of magnitude during fruit development. Lescourret *et al.* (2001) and Quilot *et al.* (2005) added genetic variation by allowing parameters describing the fruit processes to vary between genotypes.

As the kiwifruit berry grows, much of its carbohydrate is stored as starch, and the soluble solids fraction in the fruit remains roughly constant until late in the season when it increases rapidly due to starch hydrolysation (Richardson *et al.*, 1997). This has been described by a starch sub-model model based upon earlier work of Hall *et al.* (2006). While dry weight accumulation in kiwifruit is roughly linear, the slope of the fresh-weight curve shows a marked reduction part way through the season (Richardson *et al.*, 1997), reflecting a roughly 5-fold drop in water uptake rates (Hall *et al.*, 2006). Measurements of fruit hydraulic resistance in kiwifruit (Mazzeo *et al.*, 2013) showed that the proportion of hydraulic resistance contributed by the pedicel and receptacle (where the fruit attaches to the pedicel) grows from less than 50% at 20 days after full bloom (DAFB) to over 80% from 50 DAFB. A separate pedicel component in the model is required to deal with this, as the approach of Fishman and Génard (1998) has hydraulic conductance increasing in proportion to the surface area of the fruit, and active uptake directly from the stem vasculature. Elasticity (Léchaudel *et al.*, 2007) and changes in cell wall extensibility to represent different phases of growth (Liu *et al.*, 2007) are incorporated so that the model can be used to simulate dynamics over the full growing season. Previous models of kiwifruit berry development have been descriptive in nature, focused on individual properties of the fruit in isolation from the plant as a whole. For example, Hall and McPherson (1997) describe a model for soluble solids content of the fruit, Hall *et al.* (2002) and Bebbington *et al.* (2009) describe curves for predicting harvest fresh weight, and Hall and Snelgar (2008) describe a regression model for predicting harvest fruit dry matter from seasonal temperatures. Hall *et al.* (2006) developed a descriptive model of the fruit which simulated a number of aspects of fruit quality, but this model did not incorporate any environmental factors.

The major motivation for this study was to link the various sub-models mentioned above to simulate the growth of a kiwifruit berry from soon after bloom to maturity. This model may then give insight into the importance of the various physiological processes which give rise to the observed two phase growth and what determines the final fruit dry-matter content, which is a primary fruit-quality attribute of commercial interest. Also, as the sub-models include carbohydrate and water availability as well as fruit temperature, the influence of immediate and seasonal environment upon fruit development will become clearer. This may provide guidance of management practices to mitigate adverse environmental conditions.

## Materials and methods

Values and units of variables, parameters, and constants used in the model are summarized in the Appendix.

### The Fishman and Génard model including elasticity

Details of the Fishman and Génard (1998) fruit model, extended to include elasticity, are given in the Supplementary material. Briefly, the fruit is described by a single compartment which takes up water and sugar through a composite membrane separating it from the xylem and phloem, and loses water and dry matter by transpiration and respiration. Import into the fruit from the xylem is proportional to the difference in water potentials, while mass flow from the phloem includes a reflection coefficient to include a component of symplastic flow. Sugar uptake is modelled as a parallel combination of active uptake, mass flow, and diffusion. In the original Fishman and Génard model the fruit growth was related to the plasticity of its tissues and was described at any time by its water content (*w*) and dry weight (*s*), both in grams. In order to account for diurnal variations of fruit volume, elasticity was included in the model (Léchaudel *et al.*, 2007), requiring that a full description of the fruit’s state must include a third variable, the fruit turgor (*P*_*f*_).

### Extensions to the model

For explanation of the symbols used below see the Appendix. For further details, see the Supplementary material. Equation numbers here begin at 18, as the first 17 equations necessary to implement the model are given only in the Supplementary material as described above.

#### Pedicel

**Fig. 1.**
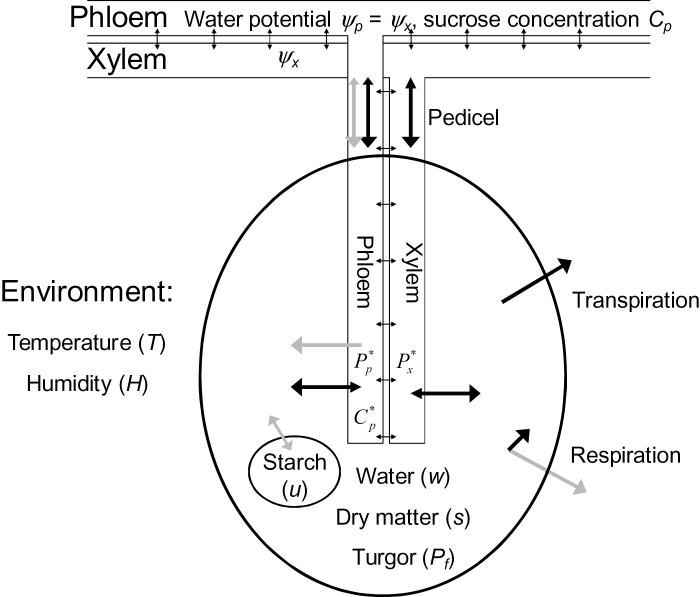
Flows of water and dry matter (sucrose equivalent) in the kiwifruit model. Black arrows indicate fluxes of water; grey arrows are fluxes of sugar or dry matter.


In kiwifruit, the pedicel/receptacle makes up a large proportion of the total hydraulic resistance of the berry (Mazzeo *et al.*, 2013). Water and sucrose solution must flow through this structure before it can be taken up by the fruit. A pedicel component is therefore added to the model (Fig. 1), and we distinguish between properties of the vasculature at the stem end of the pedicel/receptacle (*C*_*p*_, *P*_*p*_, and *π*_*p*_) and at the fruit end from which uptake processes into the fruit take place (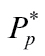,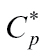, and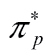). The mass flow rate through the pedicel phloem 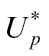 is given by

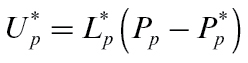
(18)

where 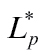 is the pedicel phloem axial conductance. Xylem flow is driven by the difference in hydrostatic pressure between the xylem in the stem and that in the fruit apoplasm, so


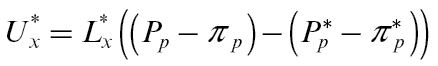
(19)

where 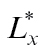 is the pedicel xylem axial conductance. We approximate the flow of sucrose through the phloem by


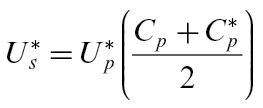
(20)

where 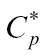 is the concentration of sugars in the fruit phloem vasculature.

Note, however, that 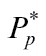 and 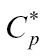 are unknown, so need to be calculated from properties of the stem and fruit. We do this by assuming that there is no storage capacity within the pedicle, so the total mass flow through the pedicel must match that taken up by the fruit,


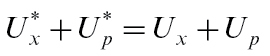
 (21)

and similarly the sucrose passing through the pedicel must match that taken up by the fruit,


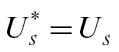
(22)

The mass-flow conservation equation 21 can be used to derive an expression for 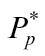 in terms of 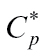 and other quantities which are known at any point in time, then the sucrose conservation equation 22 must be solved numerically for 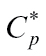.

### Starch sub-model

The original fruit model of Fishman and Génard (1998) did not include the process of sugar removal from the fruit, and hence loss of solute potential, into non-osmotically active starch. As this is an important component of early development of the kiwifruit berry, the starch sub-model of Hall *et al.* (2006), including its parameters, has been used to allocate the dry-matter content of the fruit into soluble solutes and insoluble starch. Given the dry matter (*s*) and water (*w*) content of the fruit, we first calculate *o*, the amount of dry matter that is neither starch (*u*) nor soluble solids (*s*_*s*_)


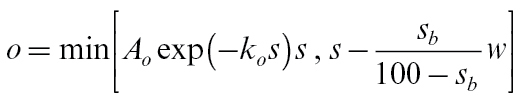
 (23)

where *A*_*o*_=0.56g, and *k*_*o*_ = 0.0384g^−1^ (values taken from Hall *et al.* 2006). That is, *o* (other dry matter, which includes cell walls, membranes, etc.) is generally an exponentially reducing proportion of dry weight (*s*), but in the early stage it may need to be limited so that the sum of dry matter of *o* and a basal soluble solids percentage needed for metabolism (*s*_*b*_=3.8%) does not exceed *s*. Then the mass of soluble solids is


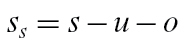
(24)

The balance between *s*_*s*_ and *u* is maintained by two rate ‘constants’, *k*_*s*_ and *k*_*u*_, describing synthesis and breakdown of starch respectively, with


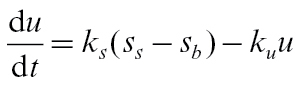
(25)

Rate *k*_*u*_ is assumed to be constant (*k*_*u*_ = 0.0551 day^−1^), but *k*_*s*_ drops with time, slowly at first then more rapidly to reach zero at time *t*_*r*_=165 DAFB (Hall *et al.* 2006);


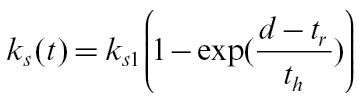
(26)

where *k*_*s*1_=0.5 day^−1^ and *t*_*h*_ = 25 days is the time before *t*_*r*_ when *k*_*s*_ drops to 63% of its maximum value. [Note there is an error in Hall *et al.* (2006) which says 73%.] The starch sub-model then allows calculation of the proportion (*Z*) of dry matter that is in soluble form


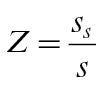
(27)

### Model parameterization for kiwifruit

To develop a model which covers both the cell division and cell expansion phases of fruit development, large changes to parameter values were required part way through the season.

#### Plastic growth parameters and elasticity

To describe growth over the entire growing season, including both cell division and cell expansion phases, cell wall extensibility *φ* must change markedly with fruit age (Liu *et al.*, 2007). We chose to describe log(*φ*) using a piecewise-linear function, with the transition between phases during *d*_1_=15 to *d*_2_=60 DAFB:


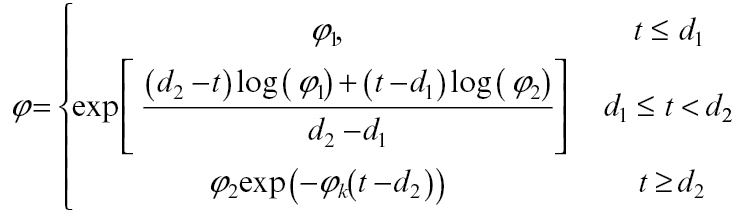
 (28)

where *φ*_1_=0.2 bar^−1^ h^−1^, *φ*_2_=0.00135 bar^−1^ h^−1^, and *φ*_k_=.0028 day^−1^. Parameter values were chosen to match the low crop-load data (see below). The yield threshold *Y* was set to a value of 2 bar, somewhat lower than the value of 5 bar used for peach in Fishman and Génard (1998), to come more into line with turgor measurements made on kiwifruit berries (Harker and Hallett 1994). Elasticity was set to 153 bar (Léchaudel *et al.*, 2007).

#### Uptake parameters

Following Fishman and Génard (1998), we made the maximum carbohydrate uptake rate *ν*_*m*_ proportional to dry weight *s*, but we also made the uptake rate dependent on temperature, so


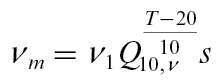
 (29)

Units for *ν*_*m*_ are [g h^−1^]; *Q*_10,*ν*_ is given the value 2 (Quinlan, 1980) and *ν*_1_ = 0.005h^−1^ is chosen to match the low crop-load data. Following Fishman and Génard (1998), we used *K*_*m*_ = 0.08, and for mass and diffusive flow from the vasculature into the fruit we set *L*_*x*_ = *L*_*p*_ = 0.0072g cm^−2^ bar^−1^, and assumed that the area of membranes grew proportionally to fruit surface area. Proportionality constants *a*_*x*_ and *a*_*p*_ (see Supplementary material) were fit to the low crop-load fresh weight data and reflection coefficients of 0.9 for phloem and 1 for xylem were assumed.

#### Pedicel conductance

Measurements by Mazzeo *et al.* (2013) show that the hydraulic resistance of the fruit decreases for the first 40 or so DAFB, then increases again before levelling off late in the season. We have approximated these measurements by fitting the following equation for pedicel xylem conductance 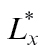 to this data,


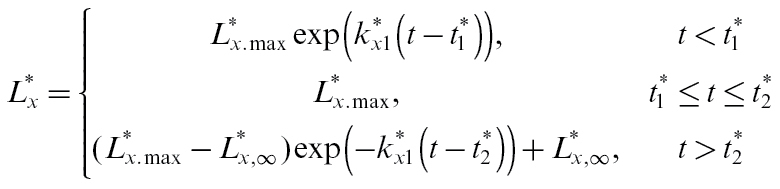
(30)

Fitted values were 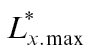
= 0.09g h^−1^ bar^−1^, 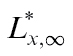
= 0.02g h^−1^ bar^−1^, 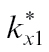
= 0.1 day^−1^, 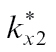
= 0.036 day^−1^, 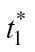
= 30 days, and 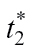
=70 days. Phloem conductance is generally about an order of magnitude lower than that of the xylem (Lacointe and Minchin, 2008). We assume that, as the pedicel is growing, phloem conductivity grows in proportion to xylem conductance. However, as there is no evidence for any breakdown in pedicel phloem functionality as the season progresses we assumed that pedicel phloem conductance then becomes constant:


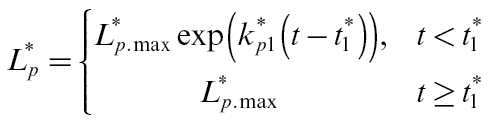
 (31)

We chose 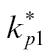
= 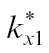
= 0.1 day^−1^ to maintain proportionality early on, and 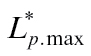
= 0.016g h^−1^ bar^−1^ to ensure reasonable fits to the low crop-load data.

#### Transpiration parameters

As in Fishman and Génard (1998), we assumed that the humidity of the air spaces within the fruit is high (0.996). Skin permeance, *ρ*, was estimated from the data of Mazzeo (2008) by fitting an equation of the form


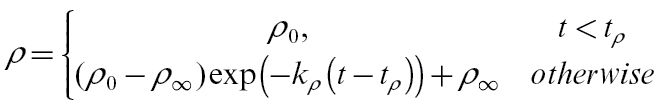
(32)

As the first data was collected 18 DAFB then followed a roughly exponential trajectory, we chose *t*_*ρ*_ = 18 days, then fitted values were *ρ*_0_=800cm h^−1^, *ρ*_*∞*_ = 25cm h^−1^, and *k*_*ρ*_ *=* 0.035 day^−1^.

Measurements made on kiwifruit berries throughout the season (data not shown) show that a good estimate of fruit surface area is given by


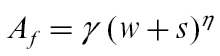
 (33)

where *γ* = 5.2076 and *η* = 0.6424.

Other parameter values, listed in the Appendix, were identical to those used in the peach model of Fishman and Génard (1998).

### Data for testing the model

Richardson *et al.* (1997) measured fruit fresh weight, dry-matter proportion (DM%), soluble solids proportion (SS%), and starch content as a proportion of dry matter (ST%) throughout the growing season on vines with high (>50 fruit m^−2^, five replicates) and low (18–30 fruit m^−2^, six replicates) crop loads. This data was collected near Whangarei, New Zealand, during the 1992/1993 season. In this study the low crop-load data was used to estimate some unknown parameters in the model, and the high crop-load data was used to test the extent to which changing the phloem sucrose concentration could adequately explain the difference between fruit development over the season.

To test the model’s ability to simulate the diurnal cycle of kiwifruit growth, we measured diameters of a number of fruit *in situ* every 10min during parts of the 2008/2009 growing season using linear voltage displacement transducers (LVDTs) at Plant and Food Research’s Te Puke research orchard. Observed diameter changes of the fruit were converted to approximate fractional changes in fruit fresh weight by assuming that fresh weight is proportional to the diameter cubed. A 5-day period, early in the growing period of relatively fine weather (days 55–60), was chosen for detailed comparison with model simulations.

### Model inputs

The fruit development model is driven by two environmental variables (temperature, *T*, and relative humidity, *H*), and two variables describing the state of the vasculature in the stem (stem water potential, *Ψ*_*x*_, and concentration of sucrose in the phloem, *C*_*p*_) at each time step. None of these inputs were directly measured by Richardson *et al.* (1997). As Whangarei does not have extensive records of hourly meteorological data, average hourly values of temperature and humidity for each month of the year were estimated instead using data from the meteorological site at Te Puke. While the Te Puke site is on average about 1.3°C cooler than Whangarei (N.Z. Meteorological Service 1983), patterns of kiwifruit development are very similar in the two locations. The diurnal patterns for both temperature and humidity could be adequately described throughout the growing season by the sine-exponential model of Parton and Logan (1981), which takes into account the changing day length. By fitting smooth second-order Fourier series to the seasonal curves of maximum and minimum temperature, and maximum and minimum humidity, a sequence of patterns suitable for describing hourly temperature and humidity on each day throughout the growing season was obtained.

The average daily pattern of stem water potential on any day of the year was estimated using


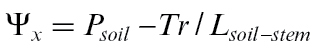
 (34)

where *Tr* is total vine transpiration (L s^−1^), the water potential of the soil *P*_*soil*_ is −1 bar for a well-watered vine, and the soil to stem water conductance *L*_*soil–stem*_ is approximately 0.001L s^−1^ bar^−1^ for kiwifruit (S. Green, personal communication). This value is at the upper end of the range of soil–stem conductance measured by Clearwater *et al.* (2004). The diurnal pattern of transpiration was approximated using a smooth curve calculated from hourly temperature, humidity, radiation, and wind using the methods of Green and McNaughton (1997). Seasonal variation in the magnitude of the transpiration curve was obtained by fitting a second-order Fourier series to monthly averages of daily transpiration calculated using the Priestly–Taylor approach.

The daily pattern of the concentration of sucrose in the phloem is unknown, but there is some evidence that maximum and minimum values do not vary markedly during the growing season (Smith and Milburn, 1980; N. Gould, personal communication). We therefore chose to set the daily minimum and maximum values for the phloem concentration to 9 and 17% as used by Fishman and Génard, but let the diurnal variation between these extrema vary in proportion to the pattern described for temperature, so that changing day length affects the average value.

**Fig. 2.**
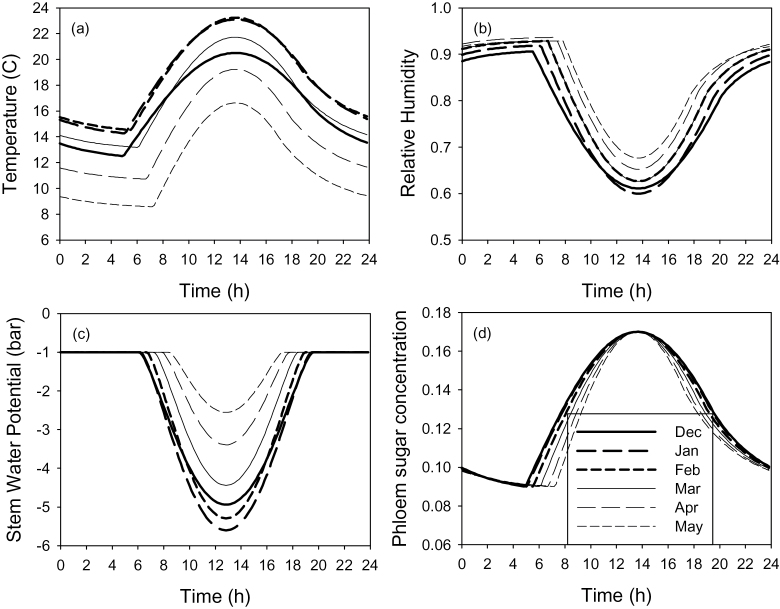
Diurnal variation in model inputs, and the variation during the growing season, for temperature (a), relative humidity (b), stem water potential (c), and phloem sucrose concentration under low crop load (d).

Model inputs reflect the large effect that seasonal variation has on temperature, humidity, and stem water potential (Fig. 2). These patterns reflect the reality that temperatures are about 7°C cooler in May than in January or February, while maximum transpiration rate is many times higher in summer than in autumn when fruit are harvested. Relative humidity shows relatively less variation between seasons, and seasonal differences in average phloem sucrose concentrations arise only from differences in daylength. Note that while humidity and temperature patterns are smoothed averages of data, the stem water potential pattern has been estimated from transpiration calculations, and the phloem sucrose pattern is simply a modification of that used by Fishman and Génard (1998).

### Model implementation

The model was implemented in SAS (version 9.2; © 2008 SAS Institute, Cary, NC, USA) as this code ran quickly, allowing rapid development and modification.

Turgor can change very rapidly from hour to hour, leading to some instability and artificial oscillations in model predictions. All simulations were therefore carried out using a fourth-order Runge–Kutta method with a half-hour time step.

### Sensitivity analysis

In order to assess the sensitivity of different aspects of the model output to changes in parameters and inputs, each parameter or input pattern was varied slightly from the parameters used to fit the Richardson *et al.* (1997) low crop-load data, and the consequent change in fresh weight, dry-matter proportion, soluble solids concentration, and starch content on day 170 (approximately harvest) were calculated.

## Results

**Fig. 3.**
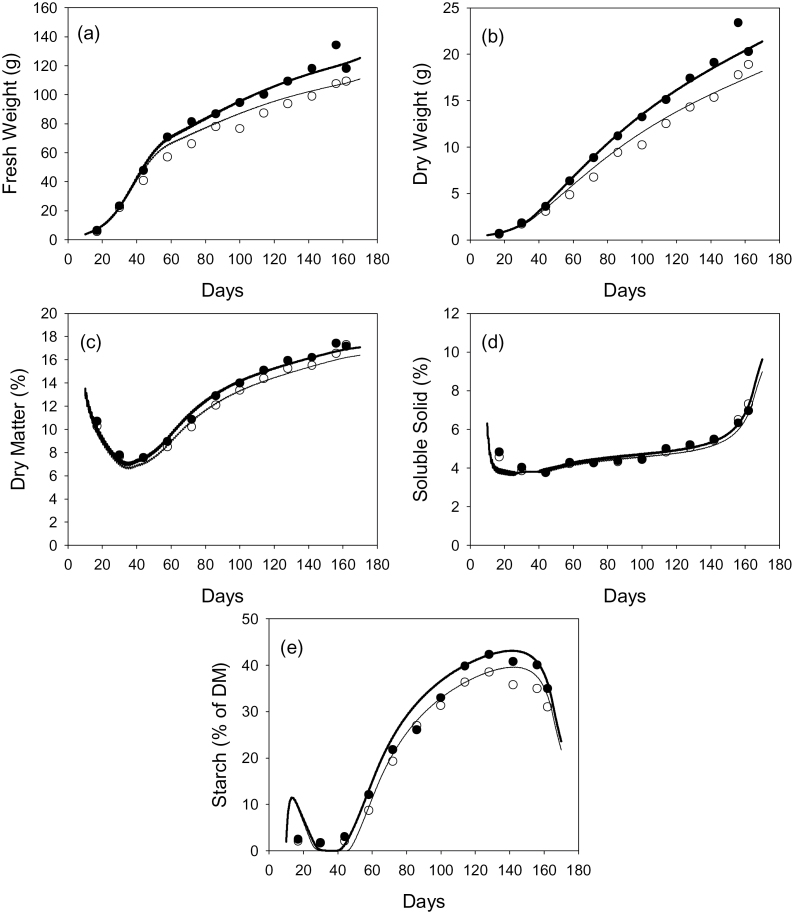
Model predictions (lines) and observed values (circles) for the development during the growing season under low (heavy line, filled circles) or high (lighter line, empty circles) crop loads, of fresh weight (a), dry weight (b), percentage of dry matter (c), percentage soluble solids (d), and starch as a percentage of dry matter (e).

The model simulates the development of fruit under low crop load well (Fig. 3, heavy lines). The addition of the pedicel compartment to the model allowed us to incorporate observed large changes in the conductivity of the pedicel/receptacle complex into the model, and thus (in concert with changes to the cell wall extensibility parameter) simulate the large change in water relations between the two major phases of kiwifruit growth (cell division and cell expansion).

Simulations for high crop load (Fig. 3, light line) were obtained by altering a single input: the concentration of sucrose in the phloem was assumed to be 1% (i.e. 0.01 *g g* ^−1^) lower throughout the season. All curves then match the high crop-load data well later in the season: fresh weight (Fig. 3a), dry weight (Fig. 3b), and percentage of dry matter in the fruit are all reduced compared with the low crop load. The starch content of the fruit is considerably lower for high crop-load fruit, but the soluble solids concentration is only marginally lower, agreeing with observed soluble solids data. In the early part of the season, however, the predictions are not so accurate: while the dry weight curve superficially seems to fit well, the fresh weight curve is a little high around day 60.

**Fig. 4.**
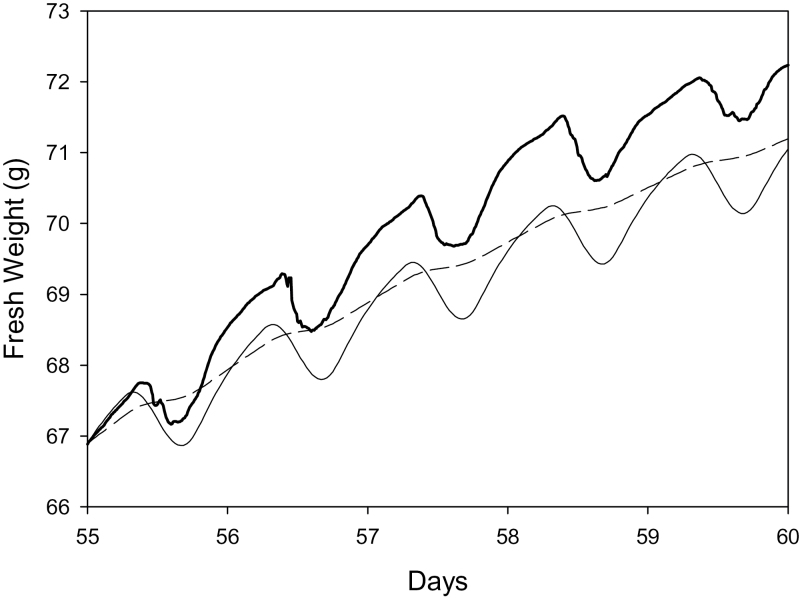
Comparison of changes in fresh weight starting from day 55 estimated from LVDT data (heavy line), the low crop-load model (light solid line), and the low crop load with no elastic component to growth and other parameters adjusted to compensate for the change (dashed line).

Fresh weights simulated by the model showed less overall growth but similar diurnal variation to that estimated by the LVDT measurements during days 55–60 (Fig. 4). Note that the LVDT data has been simply scaled to the same weight on day 55 as the model output. Despite the diurnal fluctuations in growth, no backflow through the pedicel xylem was observed in the model output (data not shown).

If elasticity is not included in the model, so that the model is more like that of Fishman and Génard (1998), then the overall growth rate is considerably higher (not shown). If parameters representing a scaling factor for the cell wall extensibility, and the area of the membrane separating the fruit from the vasculature are refitted to the low crop-load data of Richardson *et al.* (1997), reasonable fits are obtained to the long-term data of Richardson *et al.* (1997). However, the diurnal variation is then considerably less than that shown by the LVDT data (Fig. 4). An elastic component to the change in volume is clearly necessary to simulate the observed diurnal variation.

### Sensitivity analysis

#### Sensitivity to model inputs

The maximum and minimum value of each input was varied throughout the season by amounts thought to reflect expected changes between locations or seasons, and model outputs on day 170 compared with the ‘base model’ (Table 1).

**Table 1.** Sensitivity of model outputs to changes in inputs, the maximum and minimum values used each day for concentration of sugars in the phloem (*C*_*p*_), temperature (*T*), stem water potential (*P*_*x*_), and humidity (*H*)Each value for fresh weight (FW, g), dry weight (DW, g), percentage of dry matter (DM%), soluble solids percentage (SS%), and starch as a percentage of dry weight (ST%) is the average change induced by changing the input value by plus or minus the stated amount.

ID	Change	Change on day 170				
		FW	DW	DM%	SS%	ST%
*C*_*p*_ (min)	0.02	16.5	3.1	0.3	0.4	1.4
*C*_*p*_ (max)	0.02	15.4	3.6	0.8	0.7	1.9
*T* (min)	2	−2.2	0.0	0.3	0.1	0.3
*T* (max)	2	−5.0	0.2	0.8	0.4	0.7
*P*_*x*_ (min)	2	57.7	1.2	−5.8	−2.9	−3.5
*P*_*x*_ (max)	0.5	40.0	0.6	−4.7	−2.4	−2.6
*H* (min)	0.1	45.3	0.4	−5.1	−2.6	−3.3
*H* (max)	0.03	19.7	0.2	−2.4	−1.2	−1.4

Increases in carbohydrate supply throughout the season either at day or at night increases fresh weight and dry weight at harvest, but only increases the percentage of dry matter in the fruit slightly. Increased maximum or minimum temperatures decreased fresh weight, but had little effect on dry weight and increased all other fruit measures

**Fig. 5.**
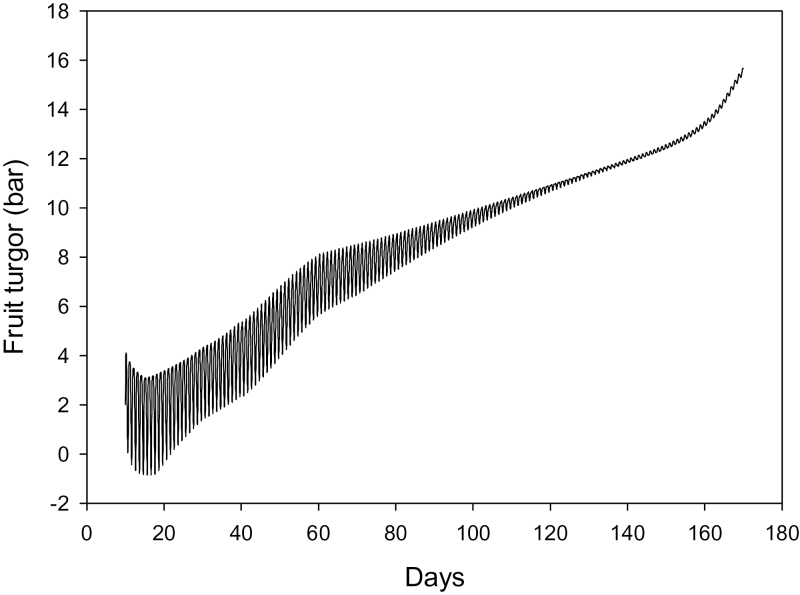
Modelled fruit turgor oscillations during the growing season. For clarity, only simulation of the low crop load is shown. Under high crop load conditions, turgor pressures are just slightly lower in the latter part of the growing season.

Predicted fruit turgor generally increases during the season, accompanied by a reduction with time in the magnitude of the diurnal variation (Fig. 5).

#### Sensitivity to model parameters

Each parameter was varied up and down, usually by 20% of the value used in the low crop-load model, and model outputs on day 170 compared with the ‘base model’ (Table 2). In some cases where the model is very insensitive to a parameter, or its value is completely unknown, a larger percentage change was used, and in other cases where parameter values were close to a ‘natural limit’ a smaller percentage change was used.

**Table 2.** Sensitivity of model outputs to changes in parameter valuesEach value for fresh weight (FW, g), dry weight (DW, g), percentage of dry matter (DM%), soluble solids percentage (SS%), and starch as a percentage of dry weight (ST%) is the average change induced by changing the parameter value by plus or minus the stated amount. Particularly large changes (FW > 16g, DW > 1g, and DM%, SS%, and ST% > 2%) are shown in bold.

Parameter group	ID	Base value	Change (%)	Change on day 170				
				FW	DW	DM%	SS%	ST%
Initial values	*w*_0_	3.2	20	11	0.2	−1.3	−0.6	−0.7
	*s*_0_	0.5	20	12	0.5	−1.3	−0.6	−0.5
	*P*_*f*0_	2	100	0	0.0	0.0	0.0	0.0
Other solutes	*π*_*p*O_	12.53	20	13	0.3	−1.6	−0.8	−0.9
	*π*_*f*O_	6.5	20	**164**	**2.8**	−**14.3**	−**7.6**	−**9.4**
Cell expansion	*Y*	0.08	25	−7	−0.1	0.0	0.0	0.2
	*d*_1_	15	20	**37**	0.6	−**4.7**	−**2.4**	−**2.5**
	*d*_2_	60	20	**181**	**2.9**	−**16.8**	−**9.3**	−**10.4**
	*φ*_1_	0.2	20	**18**	0.3	−**2.3**	−1.1	−1.2
	*φ*_2_	0.000135	20	**24**	0.4	−**3.0**	−1.5	−1.6
	*φ*_k_	0.028	20	−12	−0.2	1.4	0.7	0.7
	*ε*	153	20	−**45**	−0.7	**4.9**	**2.5**	**2.9**
Active uptake	*K*_*m*_	0.08	20	−8	−0.5	0.7	0.3	0.2
	ν_1_	0.005	20	**19**	**1.2**	−1.6	−0.7	−0.4
	*Q*_10,ν_	2	50	−11	−0.4	1.1	0.6	0.5
Membrane properties	*σ*_*p*_	0.9	5	−**32**	−0.8	**3.6**	1.8	1.8
	*a*_*x*_	0.066	20	**21**	0.3	−**2.6**	−1.3	−1.4
	*a*_*p*_	0.066	20	**36**	0.8	−**4.3**	−**2.2**	−**2.2**
	*p*_*s*_	0.003	50	5	0.3	−0.5	−0.2	−0.1
Skin permeance	*ρ*_0_	800	20	−**46**	−0.6	**5.3**	**2.7**	**3.3**
	*k*_*ρ*_	0.035	20	16	−0.1	−**2.3**	−1.2	−1.4
	*t*_*ρ*_	18	22	−**23**	−0.2	**2.9**	1.4	1.7
	*ρ*_∞_	25	20	−1	0.0	0.2	0.1	0.1
Pedicel conductance	*t*_1_*	30	20	−**52**	−0.8	**6.8**	**3.5**	**3.5**
	*t*_2_*	70	20	−1	0.5	0.6	0.3	0.6
	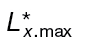	0.09	20	**25**	0.7	−**2.9**	−1.4	−1.5
	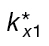	0.1	20	−**19**	−0.3	**2.3**	1.2	1.3
	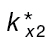	0.036	20	0	−0.3	−0.3	−0.2	−0.3
	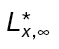	0.02	20	0	0.3	0.3	0.2	0.2
	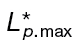	0.016	20	16	3.0	0.3	0.4	1.5
	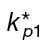	0.1	20	−5	−0.2	0.6	0.3	0.2
Starch sub-model	*u*_0_	0.01	20	0	0.0	0.0	0.0	0.0
	*A*_o_	0.56	20	−10	−0.2	1.1	0.1	−1.6
	*k*_o_	0.038	20	1	0.0	−0.2	0.3	1.7
	*s*_*b*_	3.8	20	**54**	0.9	−**5.7**	−**2.5**	−**5.6**
	*k*_*s*1_	0.5	20	−2	0.0	0.2	−0.1	1.4
	*t*_*r*_	165	6	−2	0.0	0.2	−**2.2**	**13.4**
	*t*_*h*_	25	20	0	0.0	0.0	0.2	−1.2
	*k*_*u*_	0.055	20	1	0.0	−0.2	0.5	−**3.5**

Changes to initial water and dry-matter content at the start of the simulation did have some effect on harvest fruit quality, but change in initial turgor did not. The effect of changing the osmotic pressure due to solutes other than sugar in the phloem (*π*_*p*O_) was similar to the effect of changing initial water or dry-matter contents, but the effect of changing the osmotic pressure in the fruit (*π*_*f*O_) on both fruit fresh weight and percentage of dry matter was very large. This adds directly to the turgor in the fruit (Fig. 5) so directly affects inflow rates.

Parameters controlling the relationship between cell expansion and turgor all have a significant effect, particularly on fresh weight and the percentage of dry matter in the fruit at harvest. Of the cell expansion parameters, the day when cell wall extensibility stops dropping rapidly (*d*_2_) appears to have a particularly large effect. However, it should be noted that other parameters relating to cell wall extensibility have not been measured, so the values used could possibly be in error by more than an order of magnitude. Note that the elasticity parameter also has a large effect on the size of the diurnal oscillation in fresh weight (Fig. 4). The effect of elasticity on overall seasonal development can be compensated for by altering parameters describing the membrane areas and cell wall extensibility.

The assumed reflection coefficient and area of the membrane separating fruit vasculature from the fruit cells both have significant effects on fruit development. Note that the area parameters have been fitted here to the low crop-load data. The solute permeability coefficient of the membrane has only a small effect, reflecting the small role played by diffusion of solutes through the membrane.

Parameters controlling the variation in skin permeance with fruit age, which were fitted to independent data, significantly affect the harvest fruit quality. Parameters describing receptacle conductance have a similarly large effect. The xylem conductance parameters have been fitted to independent data, but parameters controlling phloem conductance have been simply chosen to give a good fit to the low crop-load data. Remember it has been assumed that during early pedicel development, phloem and xylem conductance grow in concert, but later in the season, when xylem conductivity drops, phloem conductivity is assumed to remain constant.

Many of the parameters of the starch sub-model from Hall *et al.* (2006) primarily affected the outputs reflecting the balance between soluble and insoluble carbohydrates (SS% and ST%), as might be expected. However, harvest fresh weight was particularly sensitive to minimum level of soluble solids (*s*_*b*_).

## Discussion

By extending the model of Fishman and Génard (1998), and including a number of features from other papers we have been able to simulate kiwifruit berry carbohydrate and water dynamics over much of the growing season. With appropriate choice of parameters, the model simulated a range of time series realistically, including the complex development of DM% in the fruit (Fig. 3c), which has not been previously explained. The inclusion of an elastic component of growth as described by Léchaudel *et al.* (2007) enabled the model to simulate realistic diurnal variation in fruit size (Fig. 4) and confirmed the findings of Léchaudel *et al.* (2007) of the importance of an elastic component. Modification of the cell wall extensibility with time using the approach of Liu *et al.* (2007) allowed simulation of the entire growing season. The large contribution of the pedicel/receptacle to the hydraulic resistance of the kiwifruit berry required the inclusion of this feature in the model.

The model presented here is of the fruit alone, not of the vine as a whole. Hence, we required two inputs which are properties of the whole vine: stem water potential and the concentration of sugar in the phloem. For stem water potential, our use of the approach of Green and McNaughton (1997) ensured that values are reasonable, but for phloem sugar concentrations we have simply used the maximum and minimum daily values suggested by Fishman and Génard (1998), so the patterns used are speculative.

Although the model simulates measured patterns of fruit development well, it seems to require unrealistically high turgor within the fruit later in the season to do so (Fig. 5). Simulations using a reduced reflection coefficient *σ*_*p*_ late in the season did not reduce this turgor (data not shown). Recent work (Gould *et al.*, 2013) suggests that turgor in kiwifruit late in the season may be around an order of magnitude lower than the 15 bars suggested by the model, and turgor tends to fall, rather than rise, in ripening kiwifruit (Harker *et al.*, 1997) and other fruit (Shackel *et al.*, 1991; Matthews and Shackel, 2005; Thomas *et al.*, 2006, 2008). Decreasing turgor in ripening fruit may be associated with a rise in apoplasmic solute concentration (Wada *et al.*, 2008; Wada *et al.*, 2009), an aspect of fruit development not included here because current versions of the model do not include a separate compartment for the fruit apoplasm.

Very early in the season the model’s prediction of turgor at some times of day becomes slightly negative. This occurs during the same period when the starch sub-model predicts that starch contents are too high, and soluble solids concentrations are much lower than those observed (Fig. 3). It is likely that improvements to the starch sub-model to more realistically simulate this early stage would avoid predictions of negative turgor.

Modelled fresh and dry weight accumulation rates decline towards the end of the growing season (Fig. 3), without invoking the inhibition of active uptake as used in Fishman and Génard (1998). This could be in part because we have used input environmental variables that reflect the changing seasons, so by late in the growing season (May in New Zealand) temperatures have dropped considerably and average carbon concentrations slightly. However, a second reason is the very low cell wall extensibility required to prevent the uptake of water outstripping the dry-matter uptake late in the season, which would have lead to unrealistically low proportions of dry matter in the fruit (DM%). Compared to many other fruits (e.g. peach, mango, and tomato) kiwifruit have very low rates of water (or fresh weight) accumulation in the latter half of the growing season compared to the rate of accumulation of dry matter. A very low cell wall extensibility was needed to prevent excess water entering the fruit, thus leading to unrealistically high turgor in the fruit (Fig. 5), although this was probably because the model does not allow for membrane leakage and the resulting rise in apoplastic solute content now believed to occur during fruit maturation.

The sensitivity analysis gives us an idea as to the parameters for which we need to concentrate on getting better experimental values. The active uptake parameters affect both fresh and dry weight, and hence have a relatively small effect on final percentage of dry matter in the fruit. Somewhat paradoxically, increasing the maximum uptake rate *ν*_1_ proportionally increases fresh weight more than dry weight, thus actually decreasing the percentage of dry matter in the fruit at harvest.

Improvements are planned to the model in future. The starch sub-model of Hall *et al.* (2006) was included unchanged, but clearly needs some improvement. Very early in the season, the model over-estimates the starch component and under-estimates the soluble solids in the juice (Fig. 3), leading to negative turgor at some times of day (Fig. 5). A major change planned is to divide the fruit into apopolastic and symplastic compartments, replacing the single compartment of Fishman and Génard (1998). This will enable the model to deal explicitly with separate symplastic and apoplastic pathways, and with increasing membrane leakage as the fruit matures.

## Supplementary material

Supplementary material is available at *JXB* online.

A brief description of the model of Fishman and Génard (1998), extended to include elasticity following the approach of Léchaudel *et al.* (2007), is provided as supplementary material.

Supplementary Data

## Abbreviations:

DAFB: days after full bloom
LVDT: linear voltage displacement transducer.

## Appendix: values and units of variables, parameters, and constants

*(a) Model variables*

**Table:**

Variable	Units	Description
*C*_*p*_	g g ^−1^	(Input time series) Concentration of sugar in stem phloem
*Ψ*_*x*_	bar	(Input time series) Stem water potential
*T*	°C	(Input time series) Fruit temperature
*H*	–	(Input time series) Relative humidity of air
*s*	g	(State variable) dry weight of fruit
*w*	g	(State variable) water in fruit
*P*_*f*_	bar	(State variable) Hydrostatic pressure (turgor) in fruit
*A*_*f*_	cm^2^	Fruit surface area
*A*_*x*_	cm^2^	Area, membrane separating xylem vasculature from fruit
*A*_*p*_	cm^2^	Area, membrane separating phloem vasculature from fruit
*C*_*p*_*	g g ^−1^	Concentration of sugar in fruit phloem vasculature
*C*_*f*_	g g ^−1^	Concentration of sugar in fruit
*P*_*p*_	bar	Hydrostatic pressure in stem phloem
*P*_*p*_*	bar	Hydrostatic pressure in fruit phloem vasculature
*π*_*p*_	bar	Osmotic pressure in stem phloem
*π*_*p*_*	bar	Osmotic pressure in fruit phloem vasculature
*π*_*f*_	bar	Osmotic pressure in fruit
*R*_*f*_	g h^−1^	Total fruit respiration
*T*_*f*_	g h^−1^	Total fruit transpiration
*U*_*a*_	g h^−1^	Active uptake of sugar
*U*_*p*_	g h^−1^	Mass flow (water) from phloem vasculature into fruit
*U*_*x*_	g h^−1^	Mass flow (water) from xylem vasculature into fruit
*U*_*s*_	g h^−1^	Total rate of sugar uptake
*V*	cm^3^	Volume of fruit

*(b) Model parameters and constants*

**Table:**

Variable	Value	Units	Description
*w*_0_	3.2	g	(Initial value) Initial water content of fruit
*s*_0_	0.5	g	(Initial value) Initial dry weight of fruit
*P*_*f*0_	2	bar	(Initial value) Initial turgor pressure of fruit
*a*_*p*_	0.066	–	Area of phloem membrane as fraction of fruit surface area
*a*_*x*_	0.066	–	Area of xylem membrane as fraction of fruit surface area
*D*_*w*_	1	g cm^−3^	Density of water
*D*_*s*_	1.6	g cm^−3^	Density of dry matter
*H*_*f*_	0.996	–	Relative humidity of air spaces in fruit
*K*_*m*_	0.08	-	Michaelis constant for active transport
*L*_*x*_	0.00972	g cm^−2^ bar^−1^ h^−1^	Conductivity of xylem membrane for water transport
*L*_*p*_	0.00972	g cm^−2^ bar^−1^ h^−1^	Conductivity of phloem membrane for water transport
*p*_*s*_	0.0027	g cm^−2^ h^−1^	Permeability of phloem membrane for sugar diffusion
*Q*_10_	2.03	–	Q10 for maintenance respiration
*q*_*g*_	0.21	–	Growth respiration coefficient
*q*_*m*,293_	0.000131	h^−1^	Maintenance respiration coefficient
*R*	83	cm^3^ bar mol^−1^ K^−1^	Gas constant
*ν*_*m*_	(Eq. 29)	g h^−1^	Maximal rate of active sucrose uptake
*φ*	(Eq. 28)	bar^−1^ h^−1^	Cell wall extensibility (Lockhart)
*Y*	2	bar	Threshold pressure (Lockhart)
*Z*	(Eq. 27)	–	Proportion of dry weight which is soluble sugars
*ρ*	(Eq. 32)	cm h^−1^	Permeation coefficient of fruit surface to water vapour
*σ*_*p*_	0.9	–	Reflection coefficient of membrane to sugar
*γ* *η*	5.2076 0.6424		Parameters relating fruit area (cm^2^) to fresh weight (g)
*M*_*W*_	18	–	Molecular weight of water
*M*_*S*_	342.3	–	Molecular weight of sucrose
*π*_*p*o_	12.53	bar	Osmotic pressure of other solutes in phloem
*π*_*f*o_	6.5	bar	Osmotic pressure of other solutes in fruit
*L*_*p*_*	(Eq. 30)	g h^−1^ bar^−1^	Conductance of pedicel phloem
*L*_*x*_*	(Eq. 31)	g h^−1^ bar^−1^	Conductance of pedicel xylem
*ε*	153	bar	Fruit elasticity
*r*_*w*_	0.6	–	g water produced when 1g dry weight respires

Where values vary through the season, they are calculated from the equation numbers given.

## References

[CIT0001] Bebbington M Hall AJ Lai CD Zitikis R 2009 Dynamics and phases of kiwifruit (*Actinidia deliciosa*) growth curves. New Zealand Journal of Crop & Horticultural Science 37, 179–188

[CIT0002] Clearwater MJ Lowe RG Hofstee BJ Barclay C Mandemaker AJ Blattmann P 2004 Hydraulic conductance and rootstock effects in grafted vines of kiwifruit. Journal of Experimental Botany. 55, 1371–1382 15133051 10.1093/jxb/erh137

[CIT0003] Fishman S Génard M 1998 A biophysical model of fruit growth: simulation of seasonal and diurnal dynamics of mass. Plant, Cell and Environment 21, 739–752

[CIT0004] Gould N Morrison DR Clearwater MJ Ong S Boldingh HL Minchin PEH 2013 Elucidating the sugar import pathway into developing kiwifruit berries (*Actinidia deliciosa*). New Zealand Journal of Crop and Horticultural Science (in press).

[CIT0005] Green SR McNaughton KG 1997 Modelling effective stomatal resistance for calculating transpiration from an apple tree. Agricultural and Forest Meteorology 83, 1–26

[CIT0006] Hall AJ McPherson HG 1997 Predicting fruit maturation in kiwifruit (*Actinidia deliciosa*). Journal of Horticultural Science 72, 949–960

[CIT0007] Hall AJ Minchin PEH Snelgar WP 2002 Using scaled growth curves to make predictions: pitfalls and solutions. Acta Horticulturae 584, 133–139

[CIT0008] Hall AJ Richardson AC Snelgar WP 2006 Modelling fruit development in ‘Hayward’ kiwifruit. Acta Horticulturae 707, 41–47

[CIT0009] Hall AJ Snelgar WP 2008 Temperature effects on kiwifruit dry matter at harvest. Acta Horticulturae 803, 155–162

[CIT0010] Harker FR Hallett IC 1994 Physiological and mechanical properties of kiwifruit tissue associated with texture change during cool storage. Journal of the American Society for Horticultural Science 119, 987–993

[CIT0011] Harker FR Redgwell RJ Hallet IC Murray SH Carter G 1997 Texture of fresh fruit. Horticultural Reviews 20, 121–224

[CIT0012] Lacointe A Minchin PEH 2008 Modelling phloem and xylem transport within a complex architecture. Functional Plant Biology 35, 772–780 10.1071/FP08085 32688831

[CIT0013] Léchaudel M Vercambre G Lescourret F Normand F Génard M 2007 An analysis of elastic and plastic fruit growth of mango in response to various assimilate supplies. Tree Physiology 27, 219–230 17241964 10.1093/treephys/27.2.219

[CIT0014] Lescourret F Génard M Habib R Fishman S 2001 Variation in surface conductance to water vapor diffusion in peach fruit and its effects on fruit growth assessed by a simulation model. Tree Physiology 21, 735–741 11470659 10.1093/treephys/21.11.735

[CIT0015] Liu H Génard M Guichard S Bertin N 2007 Model-assisted analysis of tomato fruit growth in relation to carbon and water fluxes. Journal of Experimental Botany 58, 3567–3580 18057037 10.1093/jxb/erm202

[CIT0016] Matthews MA Shackel KA 2005 Growth and water transport in fleshy fruit. In Holbrook NM Zwieniechi MA, eds, Vascular Transport in Plants. Elsevier, London

[CIT0017] Mazzeo M 2008 Xylem transport efficiency and calcium accumulation in fruit of Actinidia deliciosa: implications for fruit quality. PhD thesis. University of Basilicata, Potenza.

[CIT0018] Mazzeo M Dichio B Clearwater MJ Montanaro G Xilioyannis C 2013 Hydraulic resistance of developing *Actinidia* fruit. Annals of Botany 112, 197–205 23658370 10.1093/aob/mct101 PMC3690990

[CIT0019] N.Z. Meteorological Service 1983 Summary of Climatological Observations to 1980. N.Z. Meteorological Service Miscellaneous Publication 177 Ministry of Transport, Wellington

[CIT0020] Parton WJ Logan JA 1981 A model for diurnal variation in soil and air temperature. Agricultural Meteorology 23, 205–216

[CIT0021] Quilot B Kervella J Génard M Lescourret F 2005 Analysing the genetic control of peach fruit quality through an ecophysiological model combined with a QTL approach. Journal of Experimental Botany 56, 3083–3092 16234283 10.1093/jxb/eri305

[CIT0022] Quinlan AV 1980 The thermal sensitivity of Michaelis-Menten kinetics as a function of substrate concentration. Journal of the Franklin Institute 310, 325–342

[CIT0023] Richardson AC McAneney KJ Dawson TE 1997 Carbohydrate dynamics in kiwifruit. Journal of Horticultural Science 72, 907–917

[CIT0024] Shackel KA Greve C Labavitch JM Ahmadi H 1991 Cell turgor changes associated with ripening in tomato pericarp tissue. Plant Physiology 97, 814–816 16668472 10.1104/pp.97.2.814 PMC1081080

[CIT0025] Smith JAC Milburn JA 1980 Osmoregulation and the control of phloem-sap composition in *Ricinus communis* L. Planta 148, 28–34 24311262 10.1007/BF00385438

[CIT0026] Thomas TR Matthews MA Shackel KA 2006 Direct in situ measurement of cell turgor in grape (*Vitis vinifera* L.) berries during development and in response to plant water deficits. Plant Cell and Environment 29, 993–1001 10.1111/j.1365-3040.2006.01496.x 17087481

[CIT0027] Thomas TR Shackel KA Matthews MA 2008 Mesocarp cell turgor in *Vitis vinifera* L. berries throughout development and its relation to firmness, growth, and the onset of ripening. Planta 228, 1067–1076 18797922 10.1007/s00425-008-0808-z

[CIT0028] Wada H Shackel KA Matthews MA 2008 Fruit ripening in *Vitis vinifera*: apoplastic solute accumulation accounts for pre-veraison turgor loss in berries. Planta 227, 1351–1361 18317799 10.1007/s00425-008-0707-3

[CIT0029] Wada H Matthews MA Shackel KA 2009 Seasonal pattern of apoplastic solute accumulation and loss of cell turgor during ripening of *Vitis vinifera* fruit under field conditions. Journal of Experimental Botany 60, 1773–1781 19386616 10.1093/jxb/erp050 PMC2671625

