# Supplementary material for: A biophysical model of kiwifruit (Actinidia deliciosa) berry development
Source: J Exp Bot. 2013 Oct 11;64(18):5473–83. doi: 10.1093/jxb/ert317 (PMC3871809; doi:10.1093/jxb/ert317)
Supplement: Supplementary Data [file supp_64_18_5473__index.html]

A biophysical model of kiwifruit (Actinidia deliciosa) berry development — A biophysical model of kiwifruit (Actinidia deliciosa) berry development — Supplementary Data 

# A biophysical model of kiwifruit (*Actinidia deliciosa*) berry development

## Supplementary Data

Data files

**Files in this Data Supplement:**

- Supplementary Data - Supplementary Data
- Supplementary Data - Supplementary Data
