# Supplementary material for: A biophysical model of kiwifruit (Actinidia deliciosa) berry development
Source: J Exp Bot. 2013 Oct 11;64(18):5473–83. doi: 10.1093/jxb/ert317 (PMC3871809; doi:10.1093/jxb/ert317)
Supplement: Supplementary Data [file supp_ert317_jexbot102210_file001.pdf]

A biophysical model of kiwifruit (*Actinidia deliciosa*) berry development.

A.J. Hall, P.E.H. Minchin, M.J. Clearwater, and M. Génard

Supplementary Data.

The Fishman and Génard model including elasticity.

The Fishman and Génard (1998) fruit model describes water and dry matter dynamics during peach fruit growth in terms of the physical forces and processes involved. The fruit is described as a single compartment, which takes up water and sugar through composite membranes separating it from the xylem and phloem, and loses water and dry matter through the processes of transpiration and respiration. The fruit's state at any time is described by two state variables, the mass of water (*w* (g)) and the dry weight (*s* (g)). The model is driven at an hourly time step by four input variables. Two of these are properties of the external environment: humidity (*H*) and temperature (*T* (^{o}C)) of the air. The other two are properties of the vasculature: the water potential of the vasculature (ψ_x (bar)) and the concentration of sugars in the phloem (C_p $(g\ g^{-1})$). It is assumed that the water potential of the phloem is the same as that of the xylem, as the separating membrane is highly permeable to water, so their hydrostatic pressures differ only due to differences in solute potentials.

In brief, the model of Fishman and Génard can be described as follows. The rates of change of fruit water (*w*) and dry matter (*s*) at any time (*t*) are given by

$$\frac{dw}{dt} = U_x + U_p + r_w R_f - T_f \qquad \text{(S1)}$$

$$\frac{ds}{dt} = U_s - R_f \qquad \text{(S2)}$$

where U_x and U_p are the amounts of water taken up per unit time from xylem and phloem respectively, U_s is the dry matter uptake rate, and T_f and R_f are total transpiration and respiration rates respectively. Note that following Brussières (1993) we have assumed that a fraction r_w=0.6 of respired dry matter is converted to water.

We denote the osmotic and hydrostatic pressures in the phloem by π_p and P_p (=π_p+ψ_x) respectively, and in the xylem by π_x and P_x. Flows pass from the vasculature through a composite membrane into the fruit tissues. The equations used to describe the mass flow through the composite membrane are the same as those used by Fishman and Génard (1998),

$$U_x = A_x L_x [P_x^* - P_f - (\pi_x^* - \pi_f)] \qquad \text{(S3)}$$

$$U_p = A_p L_p [P_p^* - P_f - \sigma_p (\pi_p^* - \pi_f)] \qquad \text{(S4)}$$

where P_f is fruit turgor, π_f is the osmotic pressure in the fruit, and a superscript * indicates values in the fruit vasculature rather than the plant vasculature in the original Fishman and Génard (1998) paper. The value of the effective reflection coefficient σ_p of the membrane separating the phloem from the fruit allows the one equation to describe both the xylem and phloem flows (i.e. equations S3 and S4 above). The osmotic pressure in the xylem vasculature π_x^* is set to zero, and as the plasma membrane is largely impermeable to sugars, a reflection coefficient of 1 is assumed for the xylem. For simplicity, Fishman and Génard (1998) assumed that the conductivity per unit area of the phloem membrane (L_p) and that of the xylem (L_x) were equal, as were the areas of the two membranes (A_p and A_x respectively). The membrane areas were assumed to be proportional to fruit surface area: $A_x = a_x A_f$, and $A_p = a_p A_f$. Uptake of sugars (and hence dry matter) from the phloem into the fruit (U_s) has three components:

$$U_s = U_a + (1 - \sigma_p) C_s U_p + A_p p_s (C_p^* - C_f) \qquad \text{(S5)}$$

The second term is uptake due to the mass flow above, and the third term is diffusive flow given a total permeability of the membrane p_s. C_p^* and C_f are the concentrations (proportions by weight) of sucrose in the phloem vasculature and fruit respectively, and C_s is the average of these two. It is assumed that a proportion Z of the dry matter s is in soluble form, i.e.

$$C_f = \frac{Zs}{w + Zs} \qquad \text{(S6)}$$

Active uptake U_a is described by Michaelis-Menten kinetics,

$$U_a = \frac{v_m C_v}{(K_M + C_v)} \qquad \text{(S7)}$$

where v_m is the maximum uptake rate (g h^{-1}) and K_M the Michaelis constant, the concentration at which the uptake rate is half its maximum. Fishman and Génard (1998) make v_m directly proportional to fruit dry weight s, and include the effect of non-competitive inhibition late in the season.

Transpiration is driven by the difference between the humidity of air spaces within the fruit (H_f = 0.996 as in Fishman and Génard, 1998) and the humidity of the air;

$$T_f = A_f \alpha \rho (H_f - H)) \qquad \text{(S8)}$$

where A_f is the surface area of the fruit, ρ is the permeation coefficient of the fruit surface to water vapour (cm h^{-1}), and α is dependent on temperature

$$\alpha = \frac{M_W P^*}{RT_{abs}} \qquad \text{(S9)}$$

$$P^* = 0.00805 \exp(0.0547T) \qquad \text{(S10)}$$

where R is the gas constant (83 cm^3 bar mol^{-1} K^{-1}), M_W is the molecular mass of water (18 g mol^{-1}), and T_{abs} (=T+273.3) is absolute temperature (K). Respiration R_f is given implicitly by

$$R_f = q_m(T)s + q_g(U_s - R_f) \qquad \text{(S11)}$$

where q_g and $q_m(T)$ are the coefficients for growth and maintenance respiration respectively, the latter being a function of temperature,

$$q_m(T) = q_{m,293} Q_{10}^{\frac{T-20}{10}} \qquad \text{(S12)}$$

with $q_{m,293}$ the value at 20 °C, (293 K) and Q_{10} the factor by which this changes for every 10 °C change in temperature.

We calculate the osmotic pressure (π) corresponding to any sucrose concentration C from

$$\pi = \pi_o + RT \frac{C}{(1-C)M_s} \qquad \text{(S13)}$$

where M_s is the molecular mass of sucrose (342.3 g mol^{-1}) and π_o is the contribution of other solutes to the osmotic pressure. Following Fishman and Génard (1998) we use π_{p0} = 12.53 bar in the phloem, and π_{f0} = 6.5 bar in the fruit).

Turgor P_f is calculated by equating two expressions for the rate of change of the volume of the fruit. Fruit volume (V) can be written simply as

$$V = \frac{w}{D_w} + \frac{s}{D_s} \quad \text{(S14)}$$

where D_w (=1) and D_s (=1.6) are the densities of water and carbohydrate respectively, so from the rate equations above

$$\frac{dV}{dt} = \frac{U_x + U_p + r_w R_f - T_f}{D_w} + \frac{U_s - R_f}{D_s} \quad \text{(S15)}$$

The second expression used in Fishman and Génard (1998) was Lockhart's equation, but we follow Léchaudel *et al.* (2007) and include elasticity, so this becomes

$$\frac{dV}{dt} = \frac{1}{\varepsilon} V \frac{dP_f}{dt} + \begin{cases} V\phi(P_f - Y) & P_f > Y \\ 0 & \text{otherwise} \end{cases} \quad \text{(S16)}$$

where Y is the yield threshold, ϕ is the cell wall extensibility, and ε is the elastic modulus or elasticity (153 bar as given by Léchaudel *et al.*, 2007). Equating the right hand side of equation S15 with the second term in equation S16 (Lockhart's equation), Fishman and Génard (1998) obtained an algebraic expression for P_f, but here we obtain the differential equation given by Léchaudel *et al.*, 2007),

$$\frac{dP_f}{dt} = \frac{\varepsilon}{V}\left[\frac{U_x + U_p + r_w R_f - T_f}{D_w} + \frac{U_s - R_f}{D_s} - \begin{cases} V\phi(P_f - Y) & P_f > Y \\ 0 & \text{otherwise} \end{cases}\right] \quad \text{(S17)}$$

This means that if elastic changes occur, then it is not possible to project the state of the fruit into the future without knowledge of its current turgor. With elasticity included, turgor P_f becomes a third state variable of the fruit, whose rate of change can be calculated from equation S17.

References

Brussières P. 1993. Potential dry matter and water import rates in the tomato fruit in relationship to fruit size. *Annals of Botany* **72,** 63-72.

Fishman S, Génard M. 1998. A biophysical model of fruit growth: simulation of seasonal and diurnal dynamics of mass. *Plant, Cell and Environment* **21,** 739-752.

Léchaudel M, Vercambre G, Lescourret F, Normand F, Génard M. 2007. An analysis of elastic and plastic fruit growth of mango in response to various assimilate supplies. *Tree Physiology* **27,** 219-230.
