# Supplementary material for: A biophysical model of kiwifruit (Actinidia deliciosa) berry development
Source: J Exp Bot. 2013 Oct 11;64(18):5473–83. doi: 10.1093/jxb/ert317 (PMC3871809; doi:10.1093/jxb/ert317)
Supplement: Supplementary Data [file supp_ert317_jexbot102210_file002.doc]

FIGURES

Figure 1.

Figure 2.

Figure 3.

Figure 4.

Figure 5.
